# CGRP-dependent sensitization of PKC-δ positive neurons in central amygdala mediates chronic migraine

**DOI:** 10.1186/s10194-022-01531-8

**Published:** 2022-12-12

**Authors:** Tse-Ming Chou, Zhung-Fu Lee, Shuu-Jiun Wang, Cheng-Chang Lien, Shih-Pin Chen

**Affiliations:** 1 Institute of Neuroscience, National Yang Ming Chiao Tung University, Taipei, 112 Taiwan; 2 Interdisciplinary Neuroscience Program, Taiwan International Graduate Program, Academia Sinica, Taipei, 115 Taiwan; 3 Brain Research Center, National Yang Ming Chiao Tung University, Taipei, 112 Taiwan; 4 Development, Disease Models and Therapeutics Graduate Program, Baylor College of Medicine, Houston, TX 77030 USA; 5 College of Medicine, College of Medicine, National Yang Ming Chiao Tung University, Taipei, 112 Taiwan; 6 Department of Neurology, Neurological Institute, Taipei Veterans General Hospital, Taipei, 112 Taiwan; 7 Institute of Clinical Medicine, National Yang Ming Chiao Tung University, Taipei, 112 Taiwan; 8 Division of Translational Research, Department of Medical Research, Taipei Veterans General Hospital, Taipei, 112 Taiwan

**Keywords:** Chronic migraine, Central amygdala, Parabrachial nucleus, CGRP, PKC-δ

## Abstract

**Background:**

To investigate specific brain regions and neural circuits that are responsible for migraine chronification.

**Methods:**

We established a mouse model of chronic migraine with intermittent injections of clinically-relevant dose of nitroglycerin (0.1 mg/kg for 9 days) and validated the model with cephalic and extracephalic mechanical sensitivity, calcitonin gene-related peptide (CGRP) expression in trigeminal ganglion, and responsiveness to sumatriptan or central CGRP blockade. We explored the neurons that were sensitized along with migraine chronification and investigated their roles on migraine phenotypes with chemogenetics.

**Results:**

After repetitive nitroglycerin injections, mice displayed sustained supraorbital and hind paw mechanical hyperalgesia, which lasted beyond discontinuation of nitroglycerin infusion and could be transiently reversed by sumatriptan. The CGRP expression in trigeminal ganglion was also upregulated. We found the pERK positive cells were significantly increased in the central nucleus of the amygdala (CeA), and these sensitized cells in the CeA were predominantly protein kinase C-delta (PKC-δ) positive neurons co-expressing CGRP receptors. Remarkably, blockade of the parabrachial nucleus (PBN)-CeA CGRP neurotransmission by CGRP_8–37_ microinjection to the CeA attenuated the sustained cephalic and extracephalic mechanical hyperalgesia. Furthermore, chemogenetic silencing of the sensitized CeA PKC-δ positive neurons reversed the mechanical hyperalgesia and CGRP expression in the trigeminal ganglion. In contrast, repetitive chemogenetic activation of the CeA PKC-δ positive neurons recapitulated chronic migraine-like phenotypes in naïve mice.

**Conclusions:**

Our data suggest that CeA PKC-δ positive neurons innervated by PBN CGRP positive neurons might contribute to the chronification of migraine, which may serve as future therapeutic targets for chronic migraine.

**Graphical Abstract:**

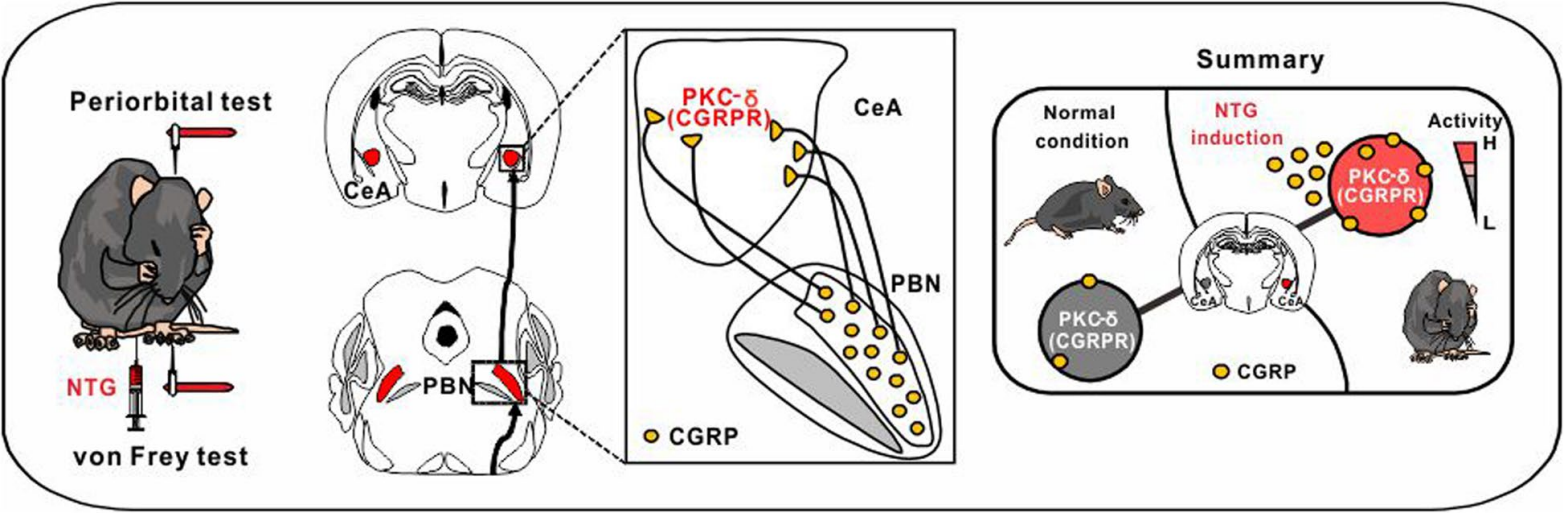

**Supplementary Information:**

The online version contains supplementary material available at 10.1186/s10194-022-01531-8.

## Background

Chronic migraine (CM) is a severe subtype of migraine which has a tremendous impact on the socioeconomic functioning and life quality of the sufferers, who account for 2% of the population worldwide [1, 2]. The diagnosis of chronic migraine requires headaches with frequency of ≥15 days per month for ≥3 months, in which ≥8 are migraine attacks or responsive to migraine-specific treatment [3]. Patients with chronic migraine are characterized by high disability, poor treatment response, frequent recurrences, high ratio of medication-overuse, and with more neuropsychiatric comorbidities. Despite the enormous clinical impact, the pathophysiological mechanisms underlying the development and chronification of migraine remain incompletely understood.

Recent studies shed light on the role of calcitonin gene-related peptide (CGRP) in the pathophysiology of migraine. CGRP levels are increased during migraine attacks and in the inter-ictal stage in patients with CM [4–7]. The Federal Drug Administration has approved multiple therapies targeting the CGRP ligands or CGRP receptors for the treatment of migraine [8, 9]. However, whether there is a central role of CGRP in migraine pathogenesis has been questioned since most of these CGRP-targeting therapies are believed to work peripherally, specifically, the dural trigeminal nociceptors and the trigeminal ganglion (TG), which are outside of blood-brain barrier [10–13]. Further, although CGRP is also believed to contribute to migraine chronification, direct evidence supporting its pathogenic role in chronic migraine remains scarce. Among the key brain regions and circuitry that have been considered important in the regulation of pain processing, the parabrachial nucleus (PBN) projection to the central nucleus of the amygdala (CeA), has been presumed relevant in migraine pathogenesis [14–16]. Remarkably, the synaptic plasticity of PBN-CeA circuit could be enhanced by perfusing CGRP in the CeA [17, 18]. Whether this CGRP-mediated neurotransmission within the PBN-CeA circuitry could contribute to migraine chronification has not been explored.

In this study, we unveiled that the PKC-δ positive neurons, co-expressed with CGRP receptors, in the CeA were sensitized during the chronification of migraine in a mouse model. These neurons fine-tuned the pain signal relayed from the CGRP-expressing neurons in the PBN and chemogenetic inhibition of these CeA PKC-δ positive neurons reversed the chronification of migraine. Moreover, repetitive chemogenetic activation of these PKC-δ positive neurons recapitulated migraine phenomenology in naïve mice. The identification of this special neuron subtype and its relevant circuitry may be pertinent for understanding the complex biology of chronic migraine.

## Methods

### Animals

The C57BL/6 J mice of either sex were used to establish the nitroglycerin-induced CM mouse model. For chemogenetic approaches, the SOM-Cre mice (stock no. 013044, The Jackson Laboratory) and PKC-δ-Cre mice (stock no. 011559, MMRRC) were used. Mice of 2–5 months old of either sex were used for all the experiments. All mice were bred in the C57BL/6 J genetic background. Mice were housed on a 12-h light-dark cycle and given food and water ad libitum. All behavioral tests were conducted after at least a 4-day habituation period. Animals were handled in accordance with the national and institutional guidelines. All behavioral procedures were conducted in accordance with the protocol approved by the Institutional Animal Care and Use Committee (IACUC) of the National Yang Ming Chiao Tung University.

### Viral vectors for chemogenetic studies

To specifically express designer receptors exclusively activated by designer drugs (DREADDs) onto SOM and PKC-δ positive neurons, a recombinant adeno-associated virus serotype 5 (rAAV5) carrying hM4Di or hM3Dq conjugated to mCherry in a double-floxed inverted open reading frame (DIO), driven by human Synapsin I (hSyn) promoter (rAAV5-hSyn-DIO-hM4Di-mCherry or rAAV5-hSyn-DIO-hM3Dq-mCherry), was used. In addition, a virus carrying the red fluorescent protein (rAAV5-hSyn-DIO-mCherry) was used as the control. All viral vectors were purchased from the Vector Core at the University of North Carolina (Chapel Hill, NC, USA) or Addgene Vector Core (Watertown, MA, USA).

### Stereotaxic virus injection

PKC-δ-Cre or SOM-Cre mice of 2–3 months old of either sex were injected with viruses in the CeA for chemogenetic studies. Mice were deeply anesthetized with isoflurane (4% induction, 1.5%–2% maintenance in oxygen, vol/vol; Halocarbon Laboratories, USA) and positioned in a stereotaxic injection frame (IVM-3000; Scientifica, Uckfield, UK). The coordinates of bilateral injection sites of the CeA were AP: − 1.31 mm, ML: ±2.87 mm, DV: − 4.82 mm. During all surgical procedures, mice were kept on a homeothermic pad (Physitemp Instrument, USA or TMP-5b, Supertech Instruments, Hungary) to maintain their body temperature at 34–37 °C. After securing the head with ear bars, the eyes of the mice were protected by the ophthalmic gel. For the virus injection, 0.35 μl/side of virus were bilaterally injected into the CeA using a 10 μl NanoFil syringe (World Precision Instruments, USA) and a 34-G beveled metal needle, controlled by the nano-pump controller (0.1 μl/min, KD Scientific, USA). After 10 min for virus distribution, the needle was withdrawn slowly. All animals were rendered to recover for at least 3 weeks before the behavioral tests for maximal viral expression.

### Stereotactic or cannula microinfusion of calcitonin gene-related peptide receptor antagonist into CeA

To block the PBN CGRP neurotransmission to the CeA, we applied the CGRP receptor 1 antagonist, CGRP fragment 8–37 (HY-P0209, MedChemExpress), dissolved in normal saline at 1.8 μg/μl [19], into bilateral CeA with either acute stereotactic microinfusion or intermittent infusion via chronically implanted cannulas. The C57BL/6 J mice of 2–3 months old were deeply anesthetized with isoflurane (4% induction, 1.5%–2% maintenance in oxygen, vol/vol; Halocarbon Laboratories, USA) and positioned in a stereotaxic injection frame (IVM-3000; Scientifica, Uckfield, UK). The coordinates of bilateral stereotactic injection or cannulas implantation sites of the CeA were AP: − 1.31 mm, ML: ±2.87 mm, DV: − 4.82 mm. For acute stereotactic application, 0.5 μl/side (900 ng/side) of CGRP_8–37_ were bilaterally injected into the CeA using a 10 μl NanoFil syringe (World Precision Instruments, USA) and a 34-G beveled metal needle, controlled by the nano-pump controller (0.1 μl/min, KD Scientific, USA). After 10 min for CGRP_8–37_ distribution, the needle was withdrawn slowly. All animals were rendered to recover for at least 30 mins before the behavioral tests. For chronic intermittent CGRP_8–37_ application, we implanted cannulas to bilateral CeA for drug infusion. After aligning, additional screws were driven onto the cranium for stabilizing the cannulas. Then, the guide cannula (62,004, OD 0.41 mm × ID 0.25 mm, RWD Life Science, China) and dummy cannula (62,104, OD 0.2 mm, RWD Life Science, China) were inserted into the designated position and the components were fixed with dental cement (Super-bond C&B kit, Sun medical, Japan). During all surgical procedures, mice were kept on a homeothermic pad (Physitemp Instrument, USA or TMP-5b, Supertech Instruments, Hungary) to maintain their body temperature at 34–37 °C. During the surgical procedures, the eyes of the mice were protected by the ophthalmic gel. All animals were rendered to recover for at least 1 week before the behavioral tests. For each injection, 0.5 μl/side (900 ng/side) of CGRP_8–37_ were bilaterally injected into the CeA using a 50 μl syringe (80,975, Hamilton, USA), controlled by the dual-channel syringe infusion pump (0.1 μl/min, Fusion 100, CHEMYX, USA). After 2 min for CGRP_8–37_ distribution, the internal cannula was withdrawn slowly.

### CTB-594 retrograde tracing

For retrograde labeling the target circuits in the central nervous system, we bilaterally injected 0.35 μl CTB-594 (C22842, Thermo, Invitrogen) into CeA using a 10 μl NanoFil syringe (World Precision Instruments, USA) and a 34-G beveled metal needle, controlled by a nano-pump controller (0.1 μl/min, KD Scientific, USA). After 10 min for CTB-594 distribution, the needle was withdrawn slowly. All animals were allowed to recover for at least 1 week before the behavioral tests.

### Nitroglycerin-induced chronic migraine model

Previously studies mostly employed nitroglycerin (NTG) with a dose of 10 mg/kg per injection to model acute or chronic migraine [20, 21]. However, this dose is 1000–10,000 times higher than that used clinically, which caused a drastic and prolonged of the blood pressure drop [22]. In contrast, a previous study has shown that a naturalistic dose of NTG, roughly 8 times higher than the reasonable pharmacological dose used clinically, is sufficient to induce allodynia in rats, without casting doubts that the animal behaviors could be confounded by the abnormal hemodynamic alterations [23, 24]. Thus, in our study, we tested multiple doses of NTG (10 mg/kg, 1 mg/kg and 0.1 kg/mg) to explore the minimal doses required to induce cranial and hindpaw allodynia in mice with least hemodynamic impact. After identifying the ideal dose (0.1 mg/kg) (see results), the mice were intraperitoneally (i.p.) injected with NTG once to mimic acute migraine or every second day for 9 days (i.e., a total of 5 NTG injections) to simulate chronic migraine. The periorbital and hindpaw mechanical thresholds of the mice were tested before and 2 hours after NTG administration on each test day. NTG solution was prepared from a stock solution of 2 mg NTG in 99% propylene glycol (#1466506, Sigma) and freshly diluted in 0.9% saline to a dose of 0.1, 1 and 10 mg/kg. The vehicle controls in these experiments contain 1% propylene glycol diluted in 0.9% saline, used as vehicle compares to that used for three different NTG doses. All injections of NTG were administered as a 0.1, 1 and 10 mg/kg volume via i.p. injection. To avoid confounding, the drugs or vehicles were prepared in a blinded fashion. To evaluate the predictive validity of the model, the migraine specific treatment sumatriptan (#S1198, Sigma), a 5-HT1B/1D agonist, was freshly dissolved in normal saline and i.p. injected at a concentration of 0.6 mg/kg, 5 mins after the NTG injection. The mechanical thresholds were only tested on the 1st, the 9th and the 10th day. These mice were similar to that in mice receiving repeated measurement in other groups [21].

### Immunohistochemistry and immunofluorescence

The mice were deeply anesthetized with isoflurane and perfused through the left ventricle to the body circulation with phosphate buffered saline (PBS, 0.9% NaCl in 0.01 M phosphate buffer, pH 7.4) followed by 4% paraformaldehyde (PFA) in the PBS. The mice brain and TG were rapidly removed and post-fixed in the 4% PFA overnight at 4 °C following previously established protocols [25–27]. Further, the solution was replaced with 15% sucrose overnight at 4 °C, followed by 30% sucrose overnight at 4 °C. The brain was then embedded in the O.C.T. solution at frozen state. Coronal brain and sagittal TG sectioning with the thickness of 50 μm [25] and 20 μm [28, 29] respectively were sliced by cryostat microtome (LEICA, CM1900, Germany) throughout the whole brain. Sections were washed with 0.1% Tween 20 in Tris-buffered saline (TBS) 5 min each free-floatingly for 3 times and then treated with 3% H_2_O_2_ in TBS for 10 min and washed by TBS again. Sections were blocked with 2% bovine serum albumin (BSA, Sigma) and 2% normal goat serum (NGS, Vector Laboratories) in TBS for 1 hour at room temperature, followed by overnight incubation with the anti-pERK1/2 antibody (1:500, #4370, Cell Signaling Technology), anti-CGRP (1:500, sc-57,053, Santa Cruz) at 4 °C and then replaced by biotinylated goat anti-rabbit antibody (1:500, Invitrogen) for 1 hour at room temperature. Further, the sections were incubated in the avidin-biotin complex reagent (ABC, Vector Laboratories). The DAB kit (Vector Laboratories) was the final step for counterstaining of the sections. For the immunofluorescence studies, the sample were overnight incubated with the anti-pERK1/2 antibody (1:500, #4370, Cell Signaling Technology), anti-CGRP (1:500, sc-57,053, Santa Cruz), anti-CALCRL (1:100, HPA008070, Sigma), anti-SOM (1:100, sc-74,556, Santa Cruz), and anti-PKC-δ (1:100, 610,398, BD Biosciences) at 4 °C and then incubated with secondary antibody Goat anti-rabbit Alexa 488 and Goat anti-mouse Alexa 594 (1:500, Invitrogen) for 2 hours at room temperature. After washing three times with TBS, slices were mounted onto slides using Vectashield mounting medium containing 4′,6-diamidino-2- phenylindole (DAPI, H-1500, Vector Laboratories,).

### Immunoblotting

The CeA isolated from brain was homogenized on ice in the RIPA buffer (Sigma) supplemented with cocktail inhibitors protease. Five microgram of protein was submitted to SDS-polyacrylamide gels 10% and transferred onto a PVDF membrane (Bio Rad). After blocking with 5% BSA, the membrane was incubated overnight at 4 C° with primary anti-pERK1/2 antibody (1:1000, #4370, Cell Signaling Technology), and anti-ERK1/2 antibody (1:1000, #9102, Cell Signaling Technology). Blots in the membrane were probed with a horseradish peroxidase coupled secondary antibody anti-mouse IgG (1:2000, #7076, Cell Signaling Technology) or anti-rabbit IgG (1:2000, #7074, Cell Signaling Technology). The enhanced chemiluminescence substrate (ECL, Pierce™ ECL Western Blotting Substrate, Invitrogen) was applied for visualization and the image was captured by luminescence imaging system (LAS-4000, Fujifilm).

### Behavioral tests

All behavioral tests were conducted in the light period of the cycle, and both male and female mice were tested in this study. Mice were transferred to the behavior room with dim light at least 30 min for habituation. Animals were randomized to experimental or control groups. For the most behavioral and biochemical tests, the experimenters were blinded and randomized to the treatment information. However, due to the specific consideration for breeding the GENSAT BAC transgenic PKC-δ-Cre mice, which should be utilized in the hemizygous state. Thus, some of the behavioral and biochemical tests of the PKC-δ-Cre mice were not randomized. In chemogenetic experiments, after a 3-week recovery from surgery, mice expressing DREADD receptors were i.p. injected with clozapine N-oxide (CNO, 5 mg/kg, Sigma). The CNO was dissolved in 0.9% NaCl with 10% dimethyl sulfoxide (DMSO). The vehicle control also contained 10% DMSO in 0.9% NaCl solution. The CNO was freshly dissolved in normal saline and i.p. injected at a concentration of 5 mg/kg for both hM4Di and hM3Dq groups. Behavioral tests were performed 1 hour (for hM4Di) [30] or 2 hours (for hM3Dq) after the drug administration. The detailed procedures of each behavioral test were as follows:

### von Frey filament test

To determine the periorbital and hind paw mechanical pain of the mice, a von Frey filament test was used. All of the von Frey tested animals were under at least a 4-day habituation period. For hind paw mechanical pain threshold measurement, mice were habituated for at least 30 min before the test. A series of von Frey filaments (0.04–1 g, Touch-Test, USA) were applied to the wire mesh onto the plantar surface of both hind paws in an up-down testing paradigm. A withdrawal response was considered valid once the hind paw removed completely from the platform. For each paw, a von Frey filament was applied five times at 5-sec intervals. The threshold was determined when paw withdrawal was observed in more than three of five applications [31]. Animals were tested for basal responses immediately before i.p. injection with NTG and vehicle control. After 75 min of the NTG and vehicle control application, animals were habituated for another 45 min, and the 2-hour post-treatment responses for mechanical sensitivity were tested. For chronic experiments, test was conducted every second day over 9 days after the drug administration (5 test days total). The test was continued for one or two further weeks without any drug administration to evaluate the sustained effect of chronic NTG treatment. As for periorbital mechanical pain measurement, the mice were habituated in the 13 × 28 × 13 cm cage 30 min before the test. A von Frey filament of 0.4 g force was applied to the periorbital area rostral to the eyes and near the midline 12 times at approximately 90° angle. The responses were recorded and scored as follows: uni- or bilateral forepaw swipes across the face (1 point), aggression/biting of the filament following stimulus (0.25 points) or clear withdrawal of the head from the stimulus (0.25 points). The points (accumulated in the 12 trials) were summed for each animal separately for each testing time to give the overall response score [32, 33].

### Marble burying test

The testing apparatus contains 6 cm depth of beddings in the 13 × 28 × 13 cm cage. Twenty-four glass marbles (about 1.5 cm of diameter) were evenly distributed on the bedding spaced 3 cm between each marble. Mice were placed individually into the testing apparatus for 30 min. Buried marbles was defined as at least two-third of their surface embedded into the bedding.

### Light/dark box (L/D box) test

After injection of NTG or vehicle drugs, mice were individually tested in a L/D box, which consists of bright and dark compartments within the same apparatus. Mice were placed individually in the middle of the light (house light, ~ 100 lx) compartment of the box and allowed to access freely to the entire apparatus for 10 min. All behaviors of mice were monitored by Tru-scan 2.0 system (Coulbourn instruments, USA). The transition between two chambers, total locomotor activity and total time spent in the two chambers were analyzed.

### Elevated Plus Maze (EPM) test

The EPM apparatus consists of two opposite open arms (30 × 5 cm) without walls surrounded by a 0.5 cm-high edge and two arms of the same dimensions enclosed by 25 cm high walls that were elevated to a height 50 cm from the floor. Animals were placed onto the center platform of the maze facing an open arm and allowed to search the maze for 10 min then return to their cages. The behavioral parameters including the percentage of time spent in both arms, the percentage of time spent in the center and total traveling distance were measured with the video tracking software EthoVision XT 13 (Noldus Information Technology, USA).

### Corticosteroid measurement

Following the behavior tests, the *submandibular* blood (cheek punch) was collected by a disposable lancet (5 mm, Goldenrold Animal Lancet, MEDIpoint, USA). In total, 0.1–0.5 ml of blood were quickly drawn without anesthesia [34]. The blood sample was centrifuged at 1900×g at 4 °C for 10 min, and the separated serum was stored at − 20 °C until further analysis. The concentration of serum corticosterone was quantified using an enzyme-linked immunoassay based commercial kit (Enzo Life Sciences, ADI-900-097, USA).

### Non-invasive blood pressure measurement

The non-invasive blood pressure sphygmomanometer for mice (NIBP System, ADINSTRUMENTS, USA), in conjunction with a data acquisition device (PowerLab System, ADINSTRUMENTS, USA) was used. This device includes a specialized tail cuff and pulse transducer, used for intermittent mouse blood pressure measurement based on the periodic occlusion of tail blood flow in unanesthetized mice. To restrain mice without anesthesia, mice were fixed to warmed stages with rodent restrainers. The baseline blood pressure was measured before and 2 hrs after the vehicle and 0.1, 1 and 10 mg/kg NTG i.p. injections.

### Statistics

The immunohistochemistry and immunofluorescence data were quantified by Image J (NIH, USA). The immunoblotting data were quantified by ImageQuant TL (GE healthcare, USA). The behavioral data were analyzed by Prism 7.0 (GraphPad Software, USA). Normality test was performed before analysis. The data were analyzed by two-way repeated measures ANOVA followed by Bonferroni post hoc test and independent t-test if they passed normality test. Otherwise, the data were analyzed with non-parametric tests such as the Friedman tests with Dunn’s post hoc test and Mann-Whitney-U test. Data were presented as mean ± SEM. Significance levels set at *p* < 0.05 *, *p* < 0.01 **, and *p* < 0.001 ***.

## Results

### Chronic nitroglycerin injection recapitulated CM-like phenomenology in mice

**Fig. 1:**
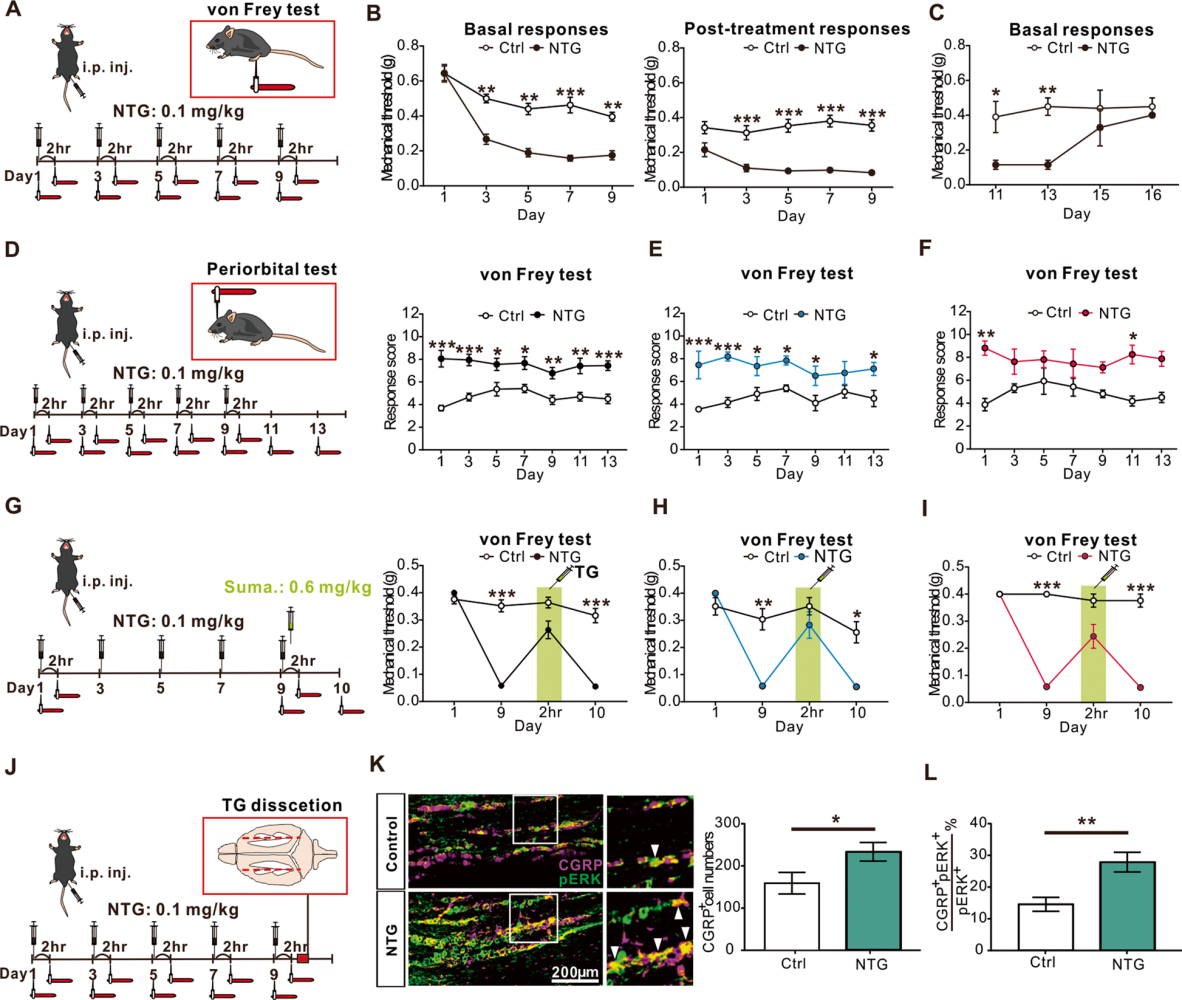
Chronic NTG injection evoked sustained CM-like phenotypes. **A** Schematic illustration of chronic NTG injection protocol. Mice of both sexes were i.p. injected with either vehicle (Ctrl) or NTG (0.1 mg/kg). **B** On day 1, the basal mechanical threshold was the same between the NTG and control group, while the formal developed mechanical hyperalgesia after 2 hrs post-treatment (left, *n* = 18 per group; *p* < 0.01 **, and *p* < 0.001 ***). Sustained basal mechanical hyperalgesia developed after repeated doses of NTG administration (right, *n* = 18 per group; *p* < 0.001 ***). **C** The basal mechanical threshold recovered 6 days after the final NTG injection (*n* = 4 per group; F(1,24) = 16.2; *p* < 0.05 *, and *p* < 0.01 **). **D** Schematic illustration of periorbital test protocol. The periorbital responses were scored 2 hrs after the i.p. injection of the NTG (left). Sustained mechanical hyperalgesia developed after repeated doses of NTG administration in both sexes of mice (right, *n* = 9 per group; F(1,112) = 117.8; *p* < 0.05 *, *p* < 0.01 **, and *p* < 0.001 ***). **E** Sustained mechanical hyperalgesia developed after repeated doses of NTG administration in male mice (*n* = 5 per group; F(1,4) = 71.6; *p* < 0.05 *, and *p* < 0.001 ***). **F** Sustained mechanical hyperalgesia developed after repeated doses of NTG administration in female mice (*n* = 4 per group; *p* < 0.05 *, and *p* < 0.01 **). **G** Schematic illustration of acute sumatriptan treatment protocol. Mice of both sexes were i.p. injected with sumatriptan (0.6 mg/kg) 5 min after NTG injection on day 9 (left). Acute sumatriptan injection transiently alleviated chronic NTG induced mechanical hyperalgesia on day 9. The basal response in the NTG group returned to mechanical hyperalgesia on day 10, suggesting that acute sumatriptan treatment only aborted acute pain but did not reverse the chronicity of mechanical hyperalgesia. (right, *n* = 20 per group; *p* < 0.001 ***). **H** Acute sumatriptan injection transiently alleviated chronic NTG induced mechanical hyperalgesia in male mice (*n* = 10 per group; *p* < 0.05 *, and *p* < 0.01 **). **I** Acute sumatriptan injection transiently alleviated chronic NTG induced mechanical hyperalgesia in female mice (*n* = 10 per group, *p* < 0.001 ***). **J** Schematic illustration of chronic NTG injection protocol. Mice were sacrificed after the NTG post-treatment von Frey test on day 9. **K** Representative images show that co-labeled CGRP and pERK neurons in the TG after chronic NTG administration were significantly higher than that in the controls (right, *n* = 4 per group; *p* = 0.043). Scale bar: 200 μm. **l** Quantification of the coloalized percentage of CGRP and pERK-positive neurons in the TG after chronic NTG induction (*n* = 4 per group; *p* = 0.003). All data shown are mean ± SEM and analyzed by Friedman tests with Dunn’s post hoc test (**B, F, G-I**) or Bonferroni post hoc test (**C-E**) or Mann-Whitney U test (**K**, **L**). Significance levels set at *p* < 0.05 *, *p* < 0.01 **, and *p* < 0.001 ***

Sustained hind paw mechanical hyperalgesia after chronic injection of NTG, a migraine trigger, is considered as a validated surrogate of CM [35–39]. As the dosage of NTG in previous studies were 1000–10,000 times higher than that used clinically, way beyond clinical and pharmacological relevance [23], we explored whether more physiological doses could still evoke sustained mechanical hyperalgesia but without compromising hemodynamics. We tried the conventional dose of NTG (10 mg/kg) and a dose with 10-fold dilution (1 mg/kg), finding that these doses could led to immediate blood pressure drop and severe stress (see Additional file 1 A-D). Two mice even died immediately after 10 mg/kg NTG injection. Hence, we further lower the dosage of NTG to 0.1 mg/kg, which is 10-fold of the dose used in human migraine model (0.5 μg/kg/min for 20 min [40]), to induce the CM-like phenotypes in mice. With chronic intermittent administration of this more clinically-relevant dose of NTG (0.1 mg/kg) every second day for 9 days (Fig. 1A), the mice of both sexes developed a sustained hind paw mechanical hyperalgesia before and 2 hours after NTG administration on each test day during the 9-day NTG injections period (Fig. 1B), similar to that observed after high doses of NTG administration (Additional file 1E, F). The sustained basal mechanical hyperalgesia lasted for another 5 days after the last NTG injection (Fig. 1C), supporting the chronicity of this model. Hence, this dose was used for all subsequent studies. To further validate the face validity of this model, we applied periorbital von Frey test (Fig. 1D) to examine the cephalic mechanical sensitivities in both male and female mice receiving repetitive NTG injection (0.1 mg/kg every second day for 9 days). Consistent with hind paw mechanical hyperalgesia, both male (Fig. 1E) and female (Fig. 1F) mice developed a sustained periorbital mechanical hyperalgesia.

We further demonstrated that i.p. injection of the migraine-specific drug sumatriptan (0.6 mg/kg) 5 min after NTG injection (0.1 mg/kg) effectively reversed the mechanical hyperalgesia transiently in both male and female mice (Fig. 1G), confirming the predictive validity of the model. Moreover, we found that sumatriptan was effective in alleviating the mechanical hyperalgesia in both male (Fig. 1H) and female (Fig. 1I) mice. We further evaluated whether these mice had adverse affective responses with approach-avoidance assay (including light/dark box and elevated plus maze) and active avoidance performance assay (i.e., marble burying test) after chronic NTG infusion. However, the trait was insignificant (Additional file 2A-D), except the marble burying test (Additional file 2B). Serum cortisol level was also similar between the NTG-injected mice and controls (Additional file 2E). Furthermore, by using phosphorylated extracellular signal-regulated kinase (pERK) immunostaining, we found that a significant proportion of the TG neurons were activated after repetitive NTG injection compared to controls, and these activated neurons were predominantly CGRP-containing neurons (Fig. 1J-L). Together, these findings supported the validities of this CM-like model.

### Central amygdala protein kinase C-delta positive neurons are sensitized in CM-like model

**Fig. 2:**
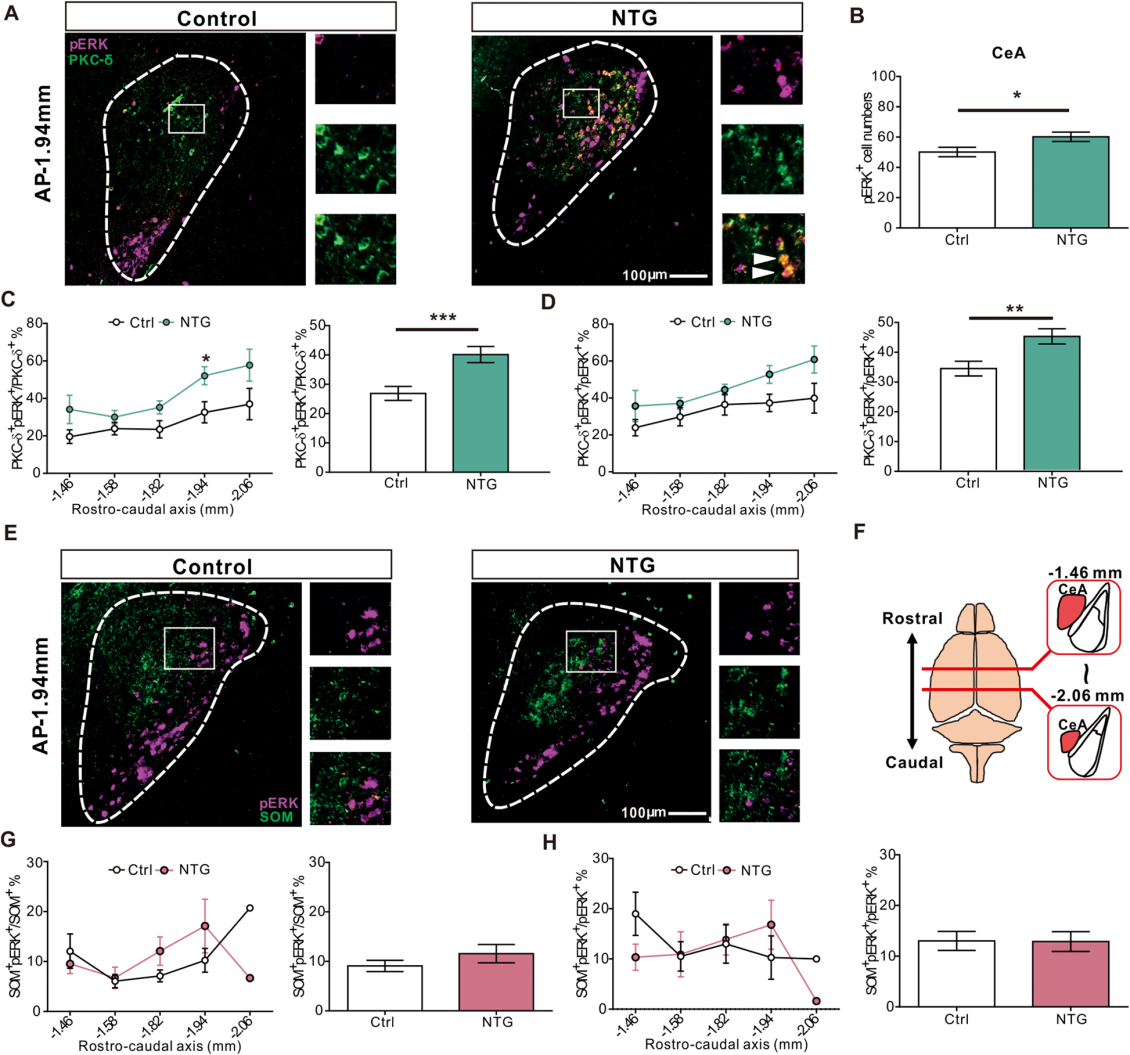
Identification and characterization of sensitized neurons in the CeA after chronification of mechanical hyperalgesia. **A** Representative low (left) and high magnification (right) images of colocalized pERK- and PKC-δ- positive neurons in the CeA. **B** After chronic NTG injection, the numbers of pERK positive neurons in the CeA were significantly higher than those in the control group (*n* = 6 per group; *p* = 0.046). Scale bar: 100 μm. **C** Rostro-caudal distribution of the percentage of pERK positive neurons colocalized with PKC-δ positive neurons in the CeA (left, *n* = 6 per group; *p* = 0.029); the percentage of pERK/PKC-δ positive neurons of the entire CeA was significantly higher in the chronic NTG treatment group than that in the control group (right, *n* = 6 per group; *p* = 0.0003). **D** Rostro-caudal distribution of the percentage of PKC-δ positive neurons colocalized with pERK positive neurons in the CeA (left, *n* = 6 per group); the percentage of PKC-δ/pERK positive neurons of the entire CeA was significantly higher in the chronic NTG treatment group than that in the control group (right, *n* = 6 per group; *p* = 0.001). **E** Representative low (left) and high magnification (right) images of colocalized pERK- and SOM- positive neurons in the CeA. Scale bar: 100 μm. **F** Schematic illustration of the rostro-caudal anatomical location of CeA relative to the position of bregma. **G** Rostro-caudal distribution of the percentage of pERK positive neurons colocalized with SOM positive neurons in the CeA (left, *n* = 5 per group). The percentage of pERK/SOM positive neurons in the entire CeA was not different between the NTG treatment and control groups (right, *n* = 5 per group; *p* = 0.597). **H** Rostro-caudal distribution of the percentage of SOM positive neurons colocalized with pERK positive neurons in the CeA (left, *n* = 5 per group). The percentage of the SOM/pERK positive neurons in the entire CeA was not different between the NTG treatment and control groups (right, *n* = 5 per group; *p* = 0.711). All data shown are mean ± SEM and analyzed Mann-Whitney U test. Significance levels set at *p* < 0.05 *, *p* < 0.01 **, and *p* < 0.001 ***

To characterize which neurons are activated following chronic NTG administration, we used pERK, which is known to induce post-translational and transcriptional regulation of molecules related to the generation of nociceptive-specific pain plasticity [41–43], to identify the activated brain regions 2 hours after the last dose of NTG injection. Across the entire brain, the pERK positive neurons were mainly identified in the CeA (Fig. 2) and paraventricular nucleus of the hypothalamus (PVN) (Additional file 3), suggesting that the neurons within these nuclei might be sensitized after chronic NTG administration. Immunoblotting further confirmed an increased phosphorylation ratio of the ERK in the CeA (Additional file 4A-C). Besides, more CeA pERK positive neurons were activated in mice receiving repetitive NTG injection compared with those receiving single injection, suggesting that more CeA neurons were sensitized along the course of migraine chronification (Additional file 4D-F). As the CeA primarily comprises heterogeneous GABAergic subpopulations of interneurons [44–46], we further investigated whether these pERK positive neurons were protein kinase C-delta (PKC-δ) or somatostatin (SOM) positive neurons, two major non-overlapping cell types in the CeA [44, 46, 47], with immunofluorescent co-labeling (Fig. 2A and E). Compared to vehicle control (Ctrl) mice, more pERK positive neurons were activated in the CeA (Fig. 2B), and the pERK was predominantly co-expressed with PKC-δ positive neurons (Fig. 2C, D) and less colocalized with SOM positive neurons (Fig. 2G, H) throughout the rostro-caudal axis of the CeA. Collectively, these data indicate that neurons sensitized by chronic NTG infusion were predominantly PKC-δ positive neurons in the CeA.

### Elevated expression of CGRP after chronic NTG administration

**Fig. 3:**
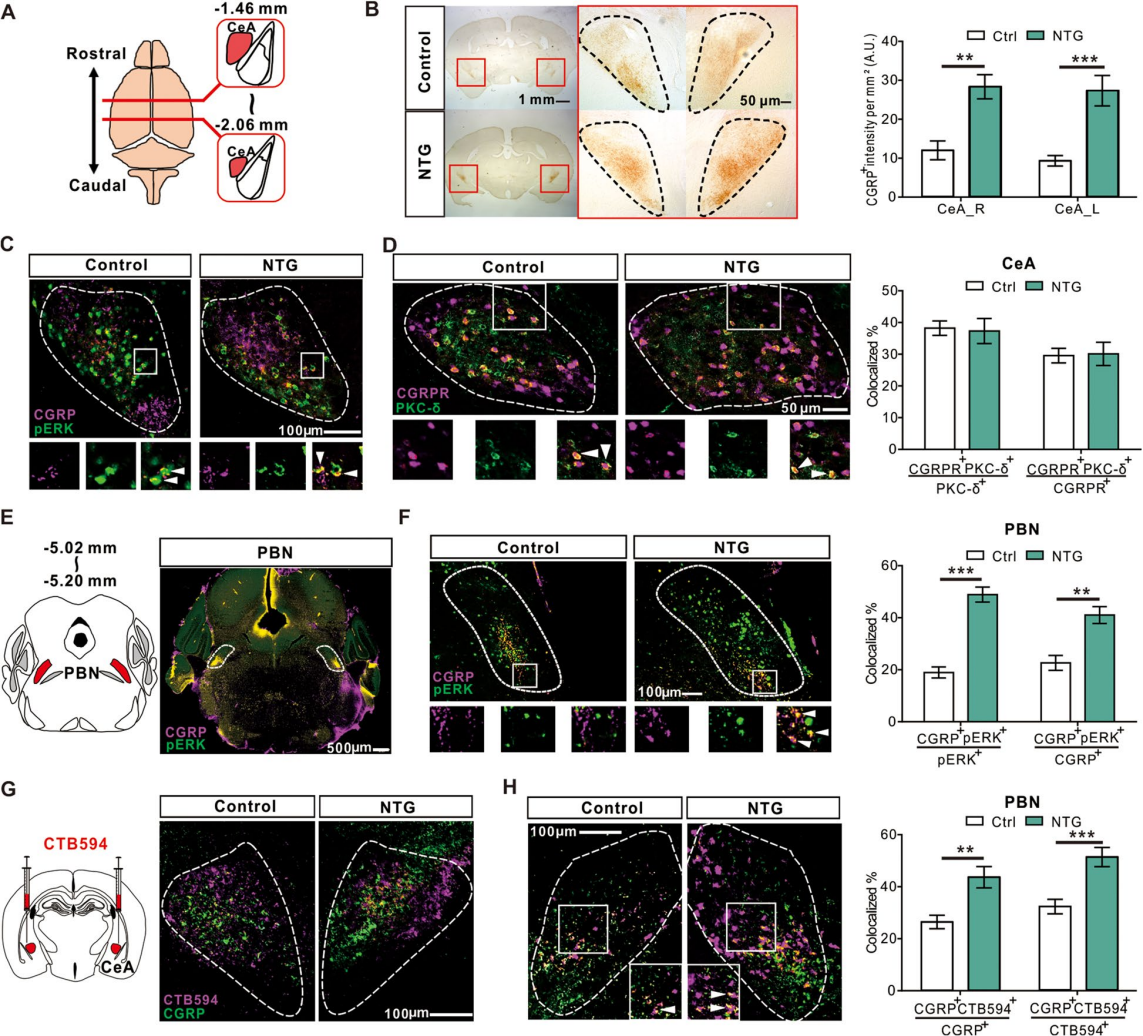
Expression of CGRP after chronic NTG administration. **A** Schematic illustration of the rostro-caudal anatomical location of CeA relative to the position of bregma. **B** Representative images of CGRP expression in the CeA after chronic NTG administration. Red dashed square indicates the high magnification of the CeA (left). The CGRP fiber intensity in the CeA after chronic NTG induction was significantly higher than that in the control group (right, *n* = 4 per group; CeA_R; *p* = 0.003; CeA_L; *p* = 0.0003). Scale bar: 1 mm (left), 50 μm (right). **C** The representative pERK positive neurons double-labeled with CGRP fibers. Scale bar: 100 μm. **D** Representative low and high magnification images of co-labeled CGRP receptor type 1 (CGRPR)- and PKC-δ- positive neurons in the CeA (left) and quantified results (right, *n* = 4 per group; CGRPR/PKC-δ; *p* = 0.593; PKC-δ/CGRPR; *p* = 0.687). Scale bar: 50 μm. **E** Representative low magnification images of co-labeled CGRP- and pERK- positive neurons in the PBN. Scale bar: 500 μm. **F** Representative low and high magnification images of co-labeled CGRP- and pERK- positive neurons in the PBN (left). The CGRP positive neurons in the PBN was significantly higher after chronic NTG administration than controls (right, *n* = 4 per group; CGRP/pERK; *p* < 0.0001; pERK/CGRP; *p* = 0.002). Scale bar: 100 μm. **G** Representative images of bilateral retrograde CTB594 labeling and co-labeled CGRP fibers in the CeA. Scale bar: 100 μm. **H** Representative images of CTB594 co-labeled CGRP positive neurons in the PBN, confirming that the CGRP positive neurons project from the PBN to CeA (left). The CGRP positive neurons in the PBN was significantly higher after chronic NTG administration than controls (right, *n* = 4 per group; CTB594/CGRP; *p* = 0.002; CGRP/CTB594; *p* = 0.0006). Scale bar: 100 μm. All data shown are mean ± SEM and analyzed by Mann-Whitney U test. Significance levels set at *p* < 0.05 *, *p* < 0.01 **, and *p* < 0.001 ***

We then checked the expression level of CGRP in the CeA by immunohistochemistry. Compared to Ctrl mice, more CGRP-containing fibers were expressed in the CeA (Fig. 3A, B). Also, the CGRP fibers were located perisomatically to pERK positive neurons within the CeA compared to Ctrl (Fig. 3C). We further found that PKC-δ- and CGRP- receptor (CGRPR) positive neurons were substantially overlapped in the caudal CeA (Fig. 3D). Besides, we also identified pERK positive neurons in the PBN, which were predominantly co-expressed with CGRP positive neurons in the NTG group (Fig. 3E, F and Additional file 5). Next, by bilateral retrograde cholera toxin subunit B-594 (CTB594) labeling, we found that the CeA CGRP fibers were originated from the CGRP positive neurons in the PBN (Fig. 3G, H). Thus, we speculated that the CGRPR-containing PKC-δ positive neurons in the CeA, innervated by the CGRP neurons in PBN, play a central role in chronic NTG-induced cephalic and extra-cephalic mechanical hyperalgesia.

### Blockade of CGRP receptors in the CeA attenuated NTG-induced mechanical hyperalgesia

**Fig. 4:**
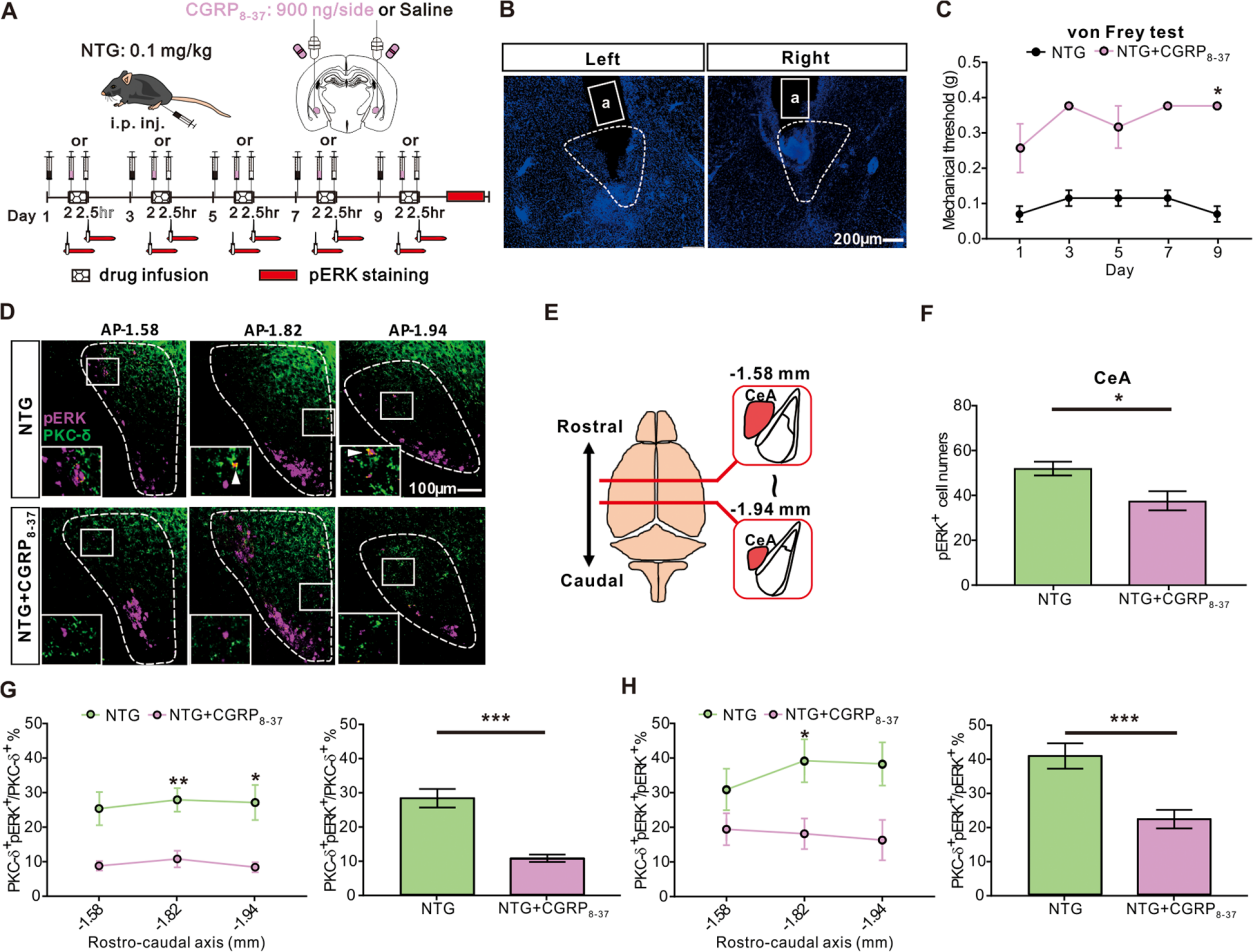
CGRPR antagonist microinfusion in the CeA. **A** Schematic illustration and the experimental timeline of bilateral CGRP_8–37_ or saline microinfusion in the CeA. **B** Representative images of the cannulas injection sites, confirming the correct injection of CGRP_8–37_ or saline at bilateral CeA. a: cannula tract. Scale bar: 200 μm. **C** Chronic CGRP_8–37_ infusion persistently alleviated NTG induced mechanical hyperalgesia (*n* = 4 per group; *p* < 0.05 *). **D** Representative images of rostral-caudal distribution of colocalized pERK- and PKC-δ- positive neurons in the CeA. Scale bar: 100 μm. **E** Schematic illustration of the rostro-caudal anatomical location of CeA relative to the position of bregma. **F** After CGRP_8–37_ infusion, the numbers of pERK positive neurons in the CeA were significantly lower than those in the saline group (*n* = 4 per group; *p* = 0.011). **G** Rostro-caudal distribution of the percentage of pERK positive neurons colocalized with PKC-δ positive neurons in the CeA (left, *n* = 4 per group; − 1.82 mm; *p* = 0.005; − 1.94 mm; *p* = 0.013); the percentage of pERK/PKC-δ positive neurons of the entire CeA was significantly lower in the CGRP_8–37_ treatment group than that in the saline group (right, *n* = 4 per group; *p* < 0.0001). **H** Rostro-caudal distribution of the percentage of PKC-δ positive neurons colocalized with pERK positive neurons in the CeA (left, *n* = 4 per group; − 1.82 mm; *p* = 0.014); the percentage of PKC-δ/pERK positive neurons of the entire CeA was significantly lower in the CGRP_8–37_ treatment group than that in the saline group (right, *n* = 4 per group; *p* = 0.0007). All data shown are mean ± SEM and analyzed by Friedman tests with Dunn’s post hoc test (**C**) or Mann-Whitney U test (**F-H**). Significance levels set at *p* < 0.05 *, *p* < 0.01 **, and *p* < 0.001 ***

To examine whether the CGRPR-containing neurons in CeA were responsible for the mechanical hyperalgesia after NTG injection, we directly applied the CGRPR antagonist, CGRP_8–37_ into bilateral CeA 2 hours after NTG administration through the cannula microinfusion every other day for 9 days (Fig. 4A, B). The mechanical hyperalgesia was attenuated after bilateral CeA cannula microinfusion of CGRP_8–37_ compared to that in the vehicle controls, strengthening the link between the sensitized CGRPR-containing neurons and behavioral phenotypes in this model (Fig. 4C). Along with the change of behavioral phenotypes, we also identified altered expression of the neurons expressing pERK and PKC-δ in the CeA after CGRP_8–37_ applications (Fig. 4D-F). The expression level of both pERK and co-expressed PKC-δ positive neurons was decreased after the CGRP_8–37_ injection compared to vehicle (Fig. 4F-H). Besides, similar results of the decreased CeA pERK positive neuron expressions after the CGRP_8–37_ injection could be found in mice receiving single (Additional file 6) and repetitive NTG injections. These results supported that the CGRPR-containing CeA PKC-δ positive neurons might play a pivotal role in NTG-induced nocifensive response.

### Chemogenetic inhibition of the CeA PKC-δ positive neurons alleviated CM-like phenotypes

**Fig. 5:**
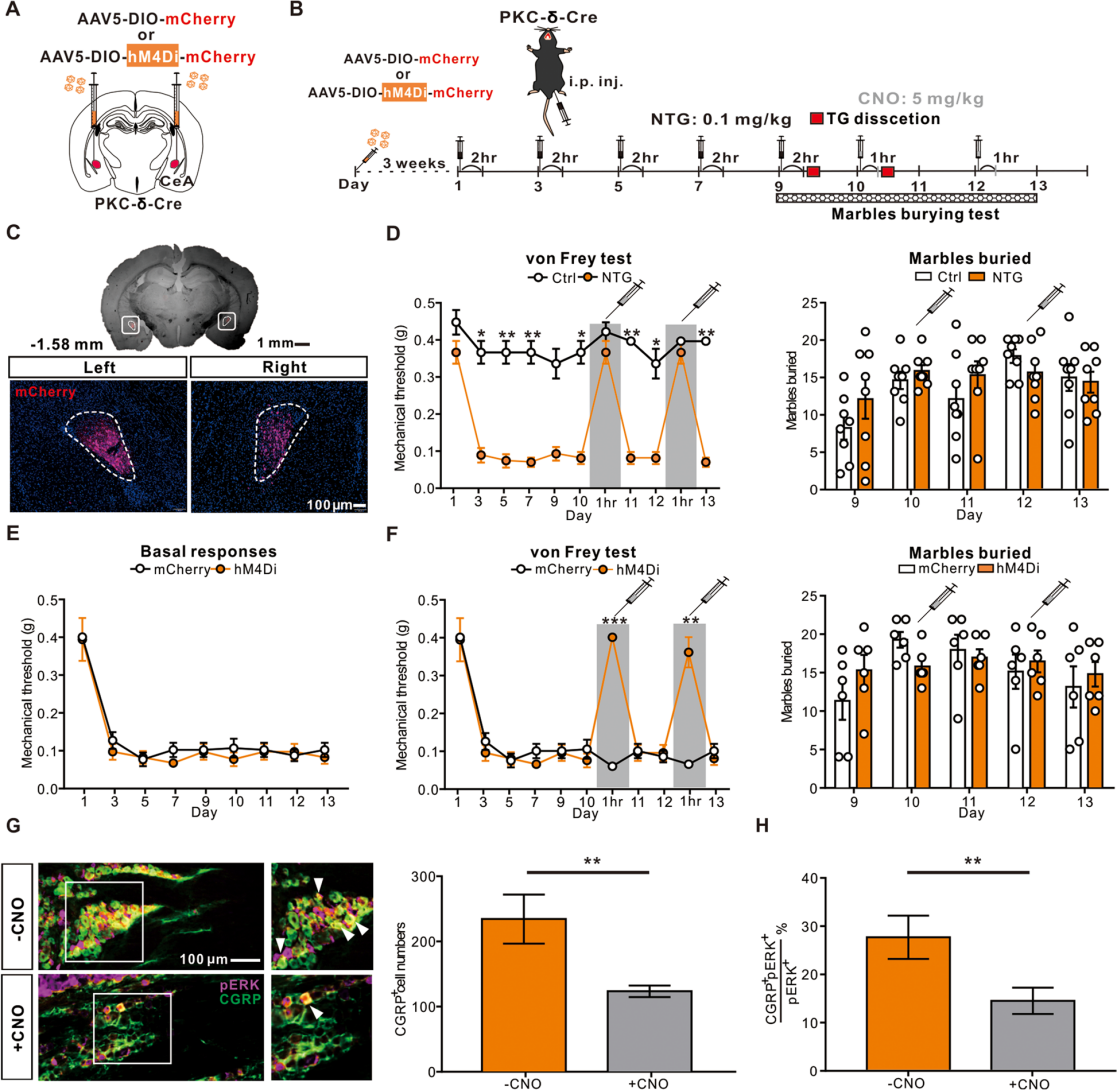
Chemogenetic inhibition of the PKC-δ positive neurons in the CeA. **A** Schematic illustration of bilateral AAV5-DIO-mCherry (mCherry) or AAV5-DIO-hM4Di-mCherry (hM4Di) injection in the CeA of PKC-δ-Cre mouse. **B** Schematic illustration of experimental timeline. After 3 weeks of virus expression, PKC-δ-Cre mice were i.p. injected with either vehicle (Ctrl) or NTG (0.1 mg/kg) every other day to day 9 (totally 5 injections) and i.p. injected with CNO (5 mg/kg) on day 10 and 12. The mechanical threshold tested 2 hrs post-treatment after the NTG injection and 1 hr. post-treatment after the CNO injection. **C** Representative low (up) and high magnification (down) images of mCherry injection sites, confirming the correct injection of viral vectors or controls at bilateral CeA. Scale bar: 100 μm. **D** Behavioral consequences before and after CNO application PKC-δ-Cre mice pretreated with bilateral hM4Di virus injection in the CeA after chronic vehicle or NTG administration. Sustained mechanical hyperalgesia alleviated 1 hr. after the CNO application on day 10 and 12 (left, *n* = 4 per group; *p* < 0.05 *, and *p* < 0.01 **) while the marble burying test was unaffected (right, *n* = 4 per group) (**E**) Sustained basal mechanical hyperalgesia developed after repeated doses of NTG administration in both mCherry and hM4Di group (*n* = 6 per group). **F** Behavioral consequences before and after CNO application PKC-δ-Cre mice pretreated with bilateral mCherry and hM4Di virus injection in the CeA and chronic NTG administration. Sustained mechanical hyperalgesia alleviated 1 hr. after the CNO application on day 10 and 12 (left, *n* = 6 per group; *p* < 0.01 **, and *p* < 0.001 ***) while the marble burying test was unaffected (right, *n* = 6 per group). (**G**) Representative images of pERK positive neurons in the TG with or without CNO application (left). The numbers of pERK positive neurons in the TG were significantly decreased after the CNO application (right, *n* = 4 per group; *p* = 0.009). Scale bar: 100 μm. **H** The percentage of CGRP/pERK positive neurons in the TG were significantly decreased after the CNO application (*n* = 4 per group; *p* = 0.005). All data shown are mean ± SEM and analyzed by Friedman tests with Dunn’s post hoc test (**D**, **F**) or Mann-Whitney U test (**G**, **H**). Significance levels set at *p* < 0.05 *, *p* < 0.01 **, and *p* < 0.001 ***

To determine whether inhibition of CeA PKC-δ positive neurons is sufficient to alleviate CM-like mechanical hyperalgesia, we injected the DREADDs, AAV5-DIO-hM4Di-mCherry, bilaterally to the CeA of PKC-δ-Cre mice (Fig. 5A and C). Three weeks after the virus injection, we repetitively injected NTG following previous paradigm to test the mechanical thresholds and anxiety-like behaviors in these mice (Fig. 5B). Supporting the role of hyper-activated CeA PKC-δ positive neurons in the pathogenesis of CM (Additional file 7), chemogenetic inhibition of the CeA PKC-δ positive neurons via clozapine N-oxide (CNO, 5 mg/kg) application on day 10 and day 12 reversed the mechanical threshold in NTG-injected mice to the level comparable to Ctrl mice (Fig. 5D left), but the anxiety-like behaviors were unaffected (Fig. 5D right). To confirm that this observation was not due to the off-target effects of CNO, we applied control AAV5-DIO-mCherry virus (mCherry) for comparison (Fig. 5A and E). Consistently, chemogenetically inhibiting the CeA PKC-δ positive neurons in hM4Di group increased the mechanical threshold comparable to the mCherry group after the CNO application (Fig. 5F left) but the anxiety-like symptoms remained unaffected (Fig. 5F right). Also, the increased CGRP (Fig. 5G, H) and PKC-δ (Additional file 8) expression level in the TG was also reversed after chemogenetically inhibiting the CeA PKC-δ positive neurons. To further address whether there are sexual differences, we applied the same protocol in both male (Additional file 9B) and female PKC-δ-Cre mice (Additional file 9C). Chemogenetically inhibiting the CeA PKC-δ positive neurons in female PKC-δ-Cre mice also reversed the mechanical threshold to that comparable to Ctrl on day 10 and day 12 (Additional file 9C). In contrast to the male mice (Additional file 9D), the female PKC-δ-Cre mice exhibited an anxiety-like behavior by burying more marbles after the NTG induction on day 9 and day 11 (Additional file 9E), which could also be reversed to the level comparable to Ctrl group after the CNO application on day 10 and day 12 (Additional file 9E). Together, these results supported our hypothesis that the sensitized CeA PKC-δ positive neurons are responsible for the sustained CM-like phenotypes.

### Chemogenetic manipulation of the CeA SOM positive neurons did not alter the mechanical threshold

**Fig. 6:**
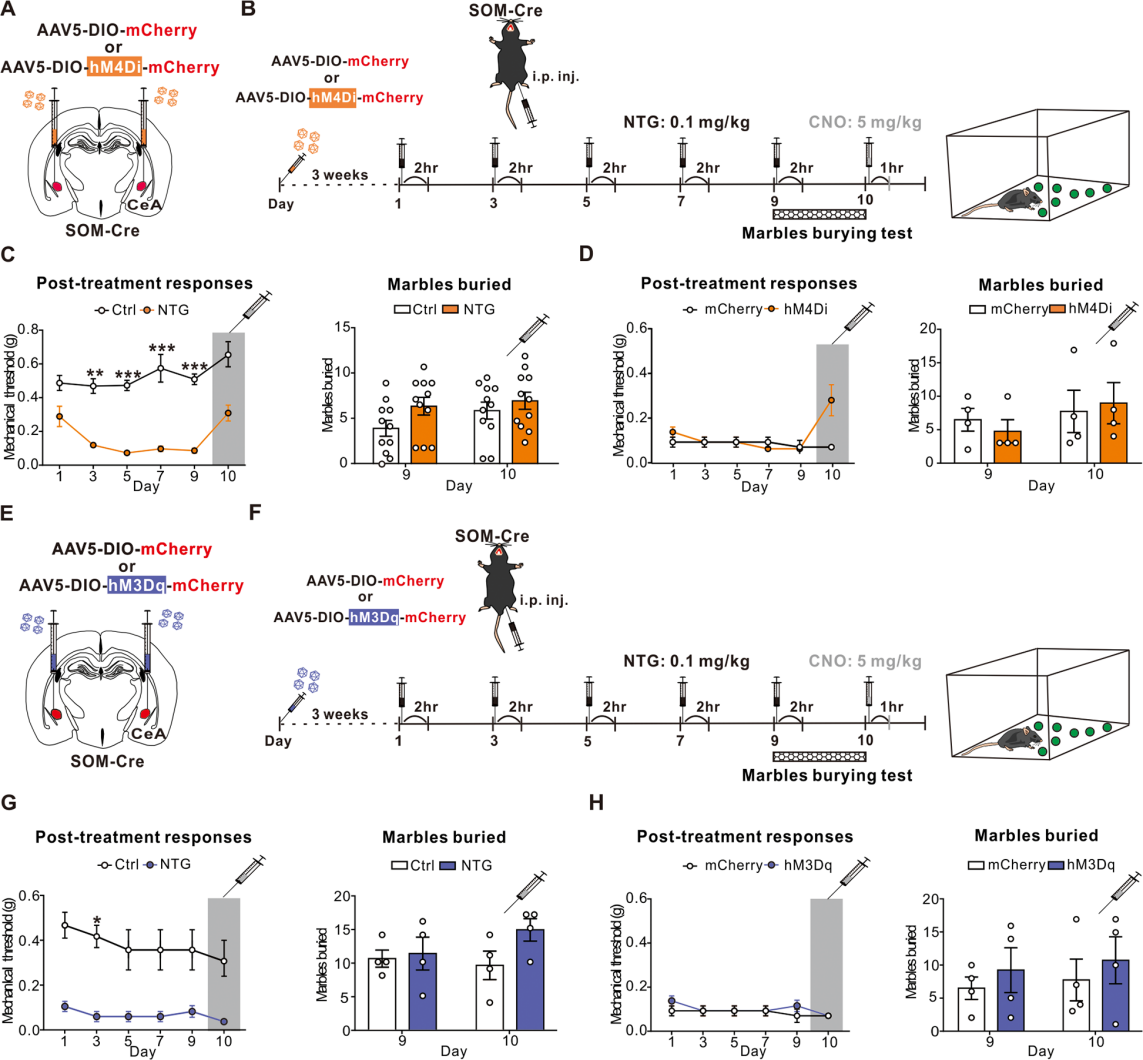
Chemogenetic manipulation of the SOM positive neurons in the CeA. **A** Schematic illustration of bilateral AAV5-DIO-mCherry (mCherry) or AAV5-DIO-hM4Di-mCherry (hM4Di) injection in the CeA of SOM-Cre mouse. **B** Schematic illustration of experimental timeline. After 3 weeks of virus expression, SOM-Cre mice were i.p. injected with either vehicle (Ctrl) or NTG (0.1 mg/kg) every other day to day 9 (totally 5 injections) and i.p. injected with CNO (5 mg/kg) on day 10 and 12. The mechanical threshold tested 2 hrs post-treatment after the NTG injection and 1 hr. post-treatment after the CNO injection. **C** Behavioral consequences before and after CNO application in SOM-Cre mice pretreated with bilateral hM4Di virus injection in the CeA and chronic NTG administration. Sustained mechanical hyperalgesia (left, *n* = 11 per group; *p* < 0.01 **, and *p* < 0.001 ***) and the marble burying test (right, *n* = 11 per group) were unaffected after chemogenetic silencing of the SOM positive neurons in CeA. **D** Behavioral consequences before and after CNO application in SOM-Cre mice pretreated with bilateral mCherry and hM4Di virus injection in the CeA and chronic NTG administration. Sustained mechanical hyperalgesia (left, *n* = 4 per group) and the marble burying test (right, *n* = 4 per group) were unaffected after chemogenetic silencing of the SOM neurons in CeA. **E** Schematic illustration of bilateral AAV5-DIO-mCherry (mCherry) or AAV5-DIO-hM3Dq-mCherry (hM3Dq) injection in the CeA of SOM-Cre mouse. **F** Schematic illustration of experimental timeline. After 3 weeks of virus expression, SOM-Cre mice were i.p. injected with either vehicle (Ctrl) or NTG (0.1 mg/kg) every other day to day 9 (totally 5 injections) and i.p. injected with CNO (5 mg/kg) on day 10 and 12. The mechanical threshold tested 2 hrs post-treatment after the NTG injection and 1 hr. post-treatment after the CNO injection. **G** Behavioral consequences before and after CNO application in SOM-Cre mice pretreated with bilateral hM3Dq virus injection in the CeA and chronic NTG administration. Sustained mechanical hyperalgesia (left, *n* = 4 per group; *p* < 0.05 *) and the marble burying test (right, *n* = 4 per group) were unaffected after chemogenetic activation of the SOM positive neurons in the CeA. **H** Behavioral consequences before and after CNO application in SOM-Cre mice pretreated with bilateral mCherry and hM3Dq virus injection in the CeA and chronic NTG administration. Sustained mechanical hyperalgesia (left, *n* = 4 per group) and the marble burying test (right, *n* = 4 per group) were unaffected after chemogenetic activation of the SOM positive neurons in the CeA. All data shown are mean ± SEM and analyzed by Friedman tests with Dunn’s post hoc test (**C**, **D**, **G, H**). Significance levels set at *p* < 0.05 *, *p* < 0.01 **, and *p* < 0.001 ***

The CeA SOM positive neurons have been shown to have opposite tuning effects on the excitability of the CeA PKC-δ positive neurons in pain modulation. Hence, we investigated whether manipulation of the CeA SOM positive neurons could alter the behavioral changes after the chronic NTG induction. Following previous protocol, we injected the inhibitory DREADDs (AAV5-DIO-hM4Di-mCherry) bilaterally to the CeA of SOM-Cre mice (Fig. 6A). Three weeks after the virus injection, we conducted repetitive NTG injection and tested the mechanical thresholds and anxiety-like behaviors in these mice (Fig. 6B). Intriguingly, the mechanical threshold and the anxiety-like behaviors were unaffected after inhibiting the CeA SOM positive neurons by CNO application (Fig. 6C). To exclude the possibility of the CNO off-target effects, we also employed control AAV5-DIO-mCherry virus (mCherry) for comparison (Fig. 6A, B). Similarly, both behaviors correlated to mechanical sensitivity and anxiety were unaffected (Fig. 6D).

We further applied the activation DREADDs (AAV5-DIO-hM3Dq-mCherry) and the control AAV5-DIO-mCherry virus (mCherry) bilaterally to the CeA of SOM-Cre mice (Fig. 6E). After 3 weeks of virus expression, we repetitively injected NTG and tested the mechanical threshold and anxiety-like behaviors in these mice (Fig. 6F). Activation of the CeA SOM positive neurons exhibited no significant difference of the mechanical threshold and the anxiety-like behaviors after the CNO application (Fig. 6G) in both NTG and mCherry control virus (Fig. 6H) groups. Taken together, these results suggest that the CeA SOM positive neurons did not contribute to CM-like mechanical hyperalgesia and the anxiety-like behaviors in this model.

### Repetitive chemogenetic activation of the CeA PKC-δ positive neurons recapitulated CM-like phenotype

**Fig. 7:**
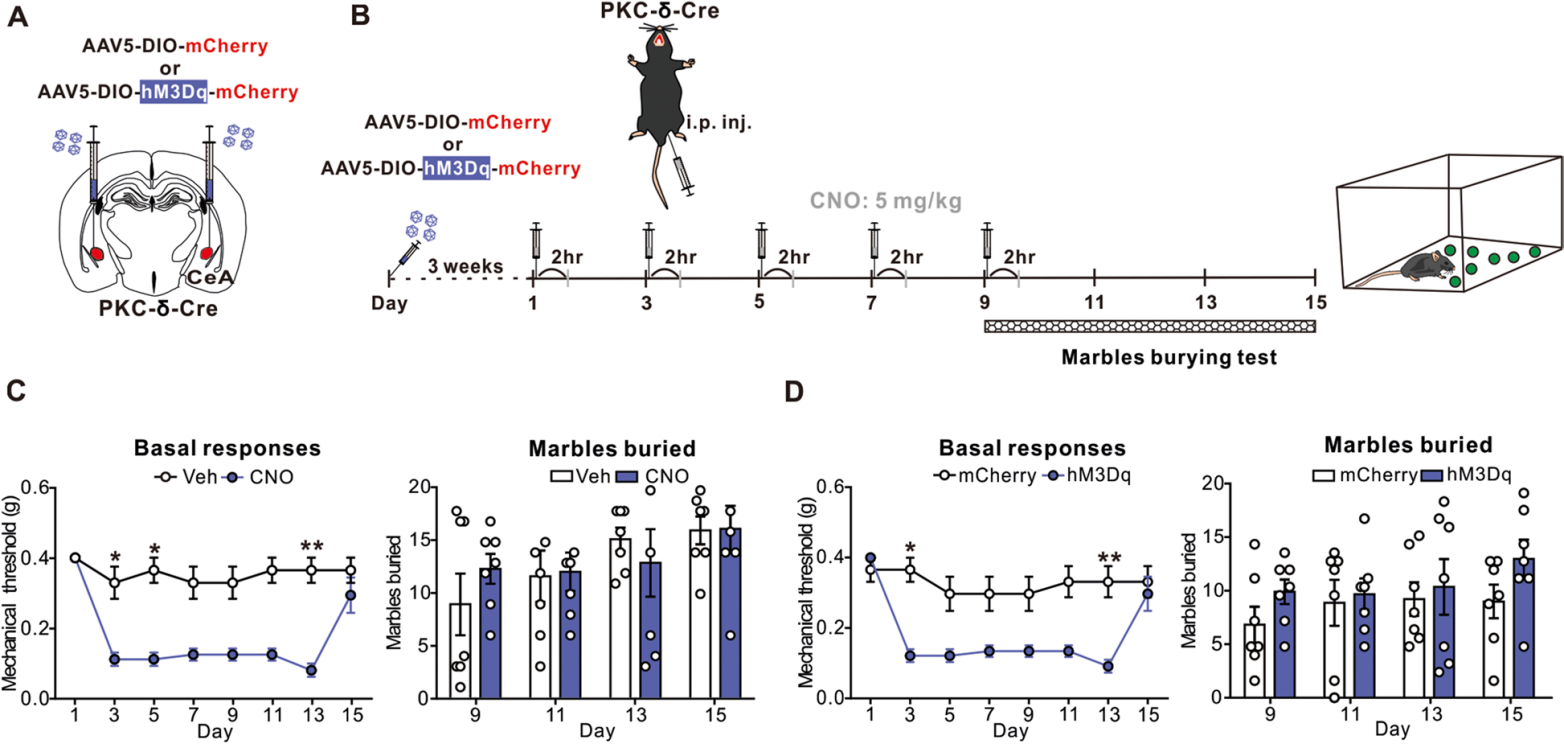
Chemogenetic activation of the PKC-δ positive neurons in the CeA. **A** Schematic illustration of bilateral AAV5-DIO-mCherry (mCherry) or AAV5-DIO-hM3Dq-mCherry (hM3Dq) injection in the CeA of PKC-δ-Cre mouse. **B** Schematic illustration of experimental timeline. After 3 weeks of virus expression, PKC-δ-Cre mice were i.p. injected with either vehicle (Veh) or CNO (5 mg/kg) every other day to day 9 (totally 5 injections) The mechanical threshold tested 2 hrs post-treatment after the CNO injection. **C** Behavioral consequences before and after CNO application in PKC-δ-Cre mice pretreated with bilateral hM3Dq virus injection in the CeA and chronic CNO administration. Both sustained mechanical hyperalgesia (left, *n* = 7 per group; *p* < 0.05 *, and *p* < 0.01 **) and unaffected marble burying test (right *n* = 7 per group) were similar to chronic NTG administration. **D** Behavioral consequences before and after CNO application in PKC-δ-Cre mice pretreated with bilateral mCherry and hM3Dq virus injection in the CeA and chronic CNO administration. Both sustained mechanical hyperalgesia (left *n* = 7 per group; *p* < 0.05 *, and *p* < 0.01 **) and unaffected marble burying test (right, *n* = 7 per group) were similar to chronic NTG administration. All data shown are mean ± SEM and analyzed by Friedman tests with Dunn’s post hoc test (**C**, **D**). Significance levels set at *p* < 0.05 *, *p* < 0.01 **, and *p* < 0.001***

To further validate whether sensitization of the CeA PKC-δ positive neurons is responsible for the chronification of migraine, we applied chemogenetic activation of the CeA PKC-δ positive neurons by repetitive infusion of CNO in naïve mice pre-treated with AAV5-DIO-mCherry or AAV5-DIO-hM3Dq-mCherry virus (Fig. 7A). Three weeks after the AAV5-DIO-mCherry or AAV5-DIO-hM3Dq-mCherry virus injection, we repetitively injected CNO using a paradigm similar to the repetitive NTG injection model and then tested the mechanical threshold and anxiety-like behaviors in these mice (Fig. 7B). Consistent with the findings in chronic NTG injection model (Fig. 1B), repetitive chemogenetic activation of the CeA PKC-δ positive neurons evoked sustained mechanical hyperalgesia (Fig. 7C left). Notably, after the final dose of CNO injection on day 9, mice exhibited sustained mechanical hyperalgesia for at least 5 days after injection (Fig. 7C left), similar to that observed in the repetitive NTG injection model (Fig. 1C), while the anxiety-like symptoms remained unaffected (Fig. 7C right). To exclude the possibility of the CNO off-target effects, we also employed control AAV5-DIO-mCherry virus (mCherry) for comparison (Fig. 7A, B). As expected, both behaviors correlated to mechanical sensitivity and the anxiety-like behaviors were unaffected (Fig. 7D). Together with the results of inactivation of the CeA PKC-δ positive neurons (Fig. 5), these data suggest that the CeA PKC-δ positive neurons might be responsible for the sensitization of chronic migraine-related mechanical hyperalgesia in a cell-type-specific manner.

## Discussion

In this study, we discovered a central cell-type-specific mechanism responsible for the chronification of migraine-like phenotypes in a chronic NTG-infusion mouse model. We found that the CGRPR-containing CeA PKC-δ positive neurons, receiving the PBN CGRP input, are sensitized during the chronification of cephalic and extracephalic mechanical hyperalgesia. Blockade of the PBN-CeA CGRP neurotransmission or chemogenetic silencing of the chronic NTG-sensitized CeA PKC-δ positive neurons could alleviate the mechanical hyperalgesia. On the contrary, repetitive activation of the CeA PKC-δ positive neurons by the application of the activation DREADDs could recapitulate the phenotypes observed in mice receiving chronic NTG application. Thus, we deemed that these CeA PKC-δ positive neurons might play a key role in CM, which may be modulated by the CGRP innervation from the PBN.

The repetitive NTG-induced CM-like model, although imperfect, is one of the most widely used models in migraine research, given the construct validity that NTG infusion also evokes migraine attack in migraineurs [35–37, 48]. However, most of the previous preclinical models employed an ultra-physiological or even potentially toxic dose way beyond the clinical and pharmacological relevance [23]. In fact, we found the mice exhibited a much longer physiological distress (data not shown) and a significant drop of blood pressure and even mortality under the dosage of 10 mg/kg (Additional file 1B-D). Thus, in this study, we used a lower dosage of NTG (0.1 mg/kg), which was approximately 10 times of the dose used in human migraine model [40] and similar to that used in a naturalistic rat model which clearly demonstrated activation of trigeminovascular system after infusion of clinically-relevant dose of NTG [23]. Under this more physiological dose, we validated the face validity of this model by showing that these mice exhibited both cephalic and extracephalic mechanical hyperalgesia (Fig. 1A-F). We also validated the predictive validity of this model by confirming that the acute migraine treatment sumatriptan can transiently alleviate the chronic NTG-induced mechanical hyperalgesia (Fig. 1G-I). In addition, we identified increased CGRP expression in the TG after repetitive NTG infusion (Fig. 1K, L), further strengthening the validity of this model. Remarkably, chemogenetic silencing of the CeA PKC-δ positive neurons could reverse these behavioral and biochemical CM-like phenotypes (Fig. 5) while repetitive chemogenetic activation of these neurons in naïve mice recapitulated similar phenotypes observed after chronic NTG infusion (Fig. 7), suggesting a potential causal relationship between these neurons and migraine chronification. Although prior studies have indicated that sensitization of TG neurons are responsible for the pain in this model [49–51], our study provides an alternative perspective that there is a cell-type specific central mechanism that regulates the trigeminal or extra-trigeminal nociceptive input in CM. Notably, specifically silencing these CeA PKC-δ positive neurons with chemogenetics, without directly intervening the TG, could still reduce the CGRP expression in the TG, which further suggests a possible top-down mechanism of these neurons in regulating pain. Although a recent study found an increased PKC-δ positive neurons in TG, which we have also identified (Additional file 8), our study was in favor of the central role of CeA PKC-δ positive neurons, particularly those receiving PBN CGRP innervation, in migraine pathogenesis.

Although the recent success of CGRP-targeting therapies strongly supports the peripheral role of CGRP, our study implicates a potential central role of CGRP in migraine chronification even though this mechanism may not be the major target of the currently available CGRP blocking therapies. In fact, in addition to peripheral actions, some preclinical studies have supported a potential central role of CGRP in the pathogenesis of migraine [13, 52]. Furthermore, we identified that the PKC-δ- and CGRPR- positive neurons were substantially overlapped in the caudal CeA after chronic NTG administration (Fig. 3D). The similar findings could be found in Han et al., 2015., which suggested that CGRPR- positive neurons in the caudal CeA directly receive pain signals from the PBN CGRP- positive neurons [53]. Although the quantification result of the PKC-δ- and CGRPR- positive neurons colocalization in chronic NTG infusion model was insignificant compared to the vehicle control group (Fig. 3D), the finding that blockade CGRPR in the CeA by CGRP_8–37_ microinfusion attenuated the mechanical hyperalgesia after NTG injection supported the crucial role of CeA CGRPR positive neurons in this model (Fig. 4C). We reasoned that the increased expression of the CGRP (Figs. 1K, L and 3B, F-H) but not the CGRPR (Fig. 3D) is the major culprit for the chronification of the disease. As CGRP can facilitate the synaptic transmission of the PBN-CeA circuit, and the action site of the CGRP is post- rather than pre-synaptic [17], it is possible that more PBN CGRP positive neurons were activated (Fig. 3F and H) to increase the probability of CGRP binding to the CGRPR after the chronic NTG application. On the other hand, it has been found that the CeA connects back to the PBN with an inhibitory reciprocal projection, which could reverse behavioral hypersensitivity in persistent pain states [54]. Thus, further studies should address the role of descending modulation in CM.

The SOM- and PKC-δ-positive neurons are two distinct, non-overlapped, and reciprocal connected neuronal populations in the CeA [44, 46, 47]. Despite the known cellular and functional heterogeneity of these two neuron subtypes, studies on how these two CeA neuronal subtypes involved in pain modulation remains elusive and controversial in different models [45, 55]. Nevertheless, their roles in migraine pathogenesis have not been explored in prior studies. A recent study proposed a function of dual opposing pain modulation within the CeA subpopulations in a mouse model of sciatic nerve cuff injury [45], which demonstrated that the CeA PKC-δ positive neurons were pronociceptive while the CeA SOM positive neurons are antinociceptive. However, in our study, while showing that the CeA PKC-δ positive neurons are pronociceptive, chemogenetic activation of the CeA SOM positive neurons did not have an antinociceptive effect (Fig. 6G and H left). We believed this could be due to the mechanistical differences within different chronic pain models, which may share similar but also recruit different circuitries in the brain.

One limitation of this study is that the slices thickness immunohistochemistry or immunofluorescence staining may not be optimal for the best imaging quality or cell counting (Additional files 10 and 11). Future studies with thinner slice thickness are needed to validate the findings. Several other studies have also explored the potential mechanisms of central sensitization in rodent models of chronic migraine. For example, Long et al., 2000 found P2X4R contributes to the central sensitization of CM by releasing brain-derived neurotrophic factor (BDNF) and promoting trigeminal nucleus caudalis (TNC) neuronal hyper-excitability [56]. Different form the pERK activation in our study, Greco et al., 2018 found a significant increase in the expression of CGRP and c-Fos genes in TG after the NTG administration [57]. In recent study Krivoshein et al., 2022 revealed a specific mechanosensitive profile of nociceptive firing in females and suggest TRPM3 channels as a potential novel candidate for the generation of migraine pain, with particular relevance to females [58]. Theses potential mechanisms combined with our findings might provide more insights for further investigation.

## Conclusions

In conclusion, we demonstrate that the CGRPR-containing CeA PKC-δ positive neurons are sensitized during the chronification of migraine, which may be contributed by the increased CGRP release from the PBN. Chemogenetic inhibition or activation of these CeA PKC-δ positive neurons correlated well with the reversal or recapitulation of the CM-like phenotypes, suggesting a critical role of these cells in migraine pathogenesis. Future studies are needed to investigate the potential clinical implications of these findings.

## Supplementary Information

**Additional file 1: Supplementary Fig. 1.** The mean arterial pressure decreased dose-dependently after the NTG injection. (A) Schematic illustration of non-invasive blood pressure test. The blood pressure was measured before and 2 hrs after the vehicle (Ctrl) and NTG (0.1, 1 and 10 mg/kg) i.p. injections. (B) The level of the mean arterial pressure was decreased dose-dependently 10 mins after the NTG (0.1, 1 and 10 mg/kg) injections (*n* = 5 per group; 0.1 vs 1 mg/kg; *p* = 0.008; 0.1 vs 10 mg/kg; *p* = 0.016). (C) The level of the mean arterial pressure was decreased dose-dependently 30 mins after the NTG (0.1, 1 and 10 mg/kg) injections (*n* = 5 per group; 0.1 vs 1 mg/kg; *p* = 0.016; 0.1 vs 10 mg/kg; *p* = 0.008; 1 vs 10 mg/kg; *p* = 0.008). (D) The level of the mean arterial pressure was decreased dose-dependently 2 hrs after the NTG (0.1, 1 and 10 mg/kg) injections (*n* = 5 per group; 1 mg/kg; *p* = 0.032; 10 mg/kg; *p* = 0.008). (E) Schematic illustration of chronic NTG injection protocol. Mice were i.p. injected with either vehicle (Ctrl) or nitroglycerin (NTG, 0.1, 1 and 10 mg/kg) on day 1, 3, 5, 7 and 9. (F) Sustained basal (left, *n* = 4 per group; F(3,96) = 102.6; *p* < 0.05 *, and *p* < 0.001 ***) and post-treatment (right, *n* = 4 per group; *p* < 0.05 *, and *p* < 0.01 **) mechanical hyperalgesia developed after repeated doses of the 0.1,1 and 10 mg/kg NTG administration for 9 days. All data shown are mean ± SEM and analyzed by Mann-Whitney-U test(B-D) or Bonferroni post hoc test (F left) or Friedman tests with Dunn’s post hoc test (F right). Significance levels set at *p* < 0.05 *, *p* < 0.01 **, and *p* < 0.001 ***.

**Additional file 2: Supplementary Fig. 2.** Insignificant trait of comorbid anxiety-like readouts after chronic NTG administration. (A) Schematic illustration of behavioral assays. The approach-avoidance assay (including L/D box and EPM) and active avoidance performance assay (i.e., marble burying test) after chronic NTG injection. (B) The mice buried more marbles after the chronic NTG injection (*n* = 14 per group; t = 2.2, df = 26; *p* < 0.05 *). (C) Insignificant differences of the transition times in the light-dark box after the chronic NTG injection (*n* = 12 per group; *p* = 0.899). (D) Insignificant differences of the time spent in the open arms of the elevated plus maze after the chronic NTG injection (*n* = 8 per group; *p* = 0.195). (E) Insignificant differences of the corticosteroid level after the chronic NTG injection (*n* = 6 per group; *p* = 0.589). All data shown are mean ± SEM and analyzed by independent t-tests (B) or Mann-Whitney-U test (C-E). Significance levels set at *p* < 0.05 *

**Additional file 3: Supplementary Fig. 3.** The increased pERK positive neuron expression in the paraventricular nucleus of hypothalamus (PVN) after the NTG injection. (A) Schematic illustration of the rostro-caudal anatomical location of PVN relative to the position of bregma. (B) Representative images of pERK positive neurons in the PVN after chronic NTG injection. Red dashed square indicates the high-magnification of the PVN (left). After chronic NTG injection, the numbers of pERK positive neurons in the PVN were significantly higher than those in the control group (right, Ctrl, *n* = 6; NTG, *n* = 8; *p* = 0.020). Scale bar: 100 μm. All data shown are mean ± SEM and analyzed by Mann-Whitney-U test. Significance levels set at *p* < 0.05 *.

**Additional file 4: Supplementary Fig. 4.** The increased phosphorylation ratio and the expression level of the CeA pERK after chronic NTG administration. (A) Schematic illustration of the CeA sampling. (B) Representative data of the pERK1/2 and ERK1/2 protein level in the CeA after chronic NTG injection. (C) After chronic NTG injection, the folds change of the phosphorylation ratio in the CeA ERK level were significantly increased (*n* = 4 per group; *p* = 0.029). (D) Representative low (right) and high magnification (left) images of rostral-caudal distribution of pERK-positive neurons in the CeA after the first (Day 1) and the fifth (Day 9) NTG injection. Scale bar: 100 μm. (E) Schematic illustration of the pERK staining. (F) After chronic NTG injection, the numbers of pERK positive neurons in the CeA were significantly higher than those in the single NTG injection on Day 1 (*n* = 4 per group; *p* = 0.039). All data shown are mean ± SEM and analyzed by Mann-Whitney-U test. Significance levels set at *p* < 0.05 *.

**Additional file 5: Supplementary Fig. 5.** The CGRP positive neuron expression in the PBN. (**A**) Representative images of rostral-caudal distribution of CGRP-positive neurons in the PBN from the Allen Institute. Scale bar: 500 μm.

**Additional file 6: Supplementary Fig. 6.** CGRPR antagonist microinjection in the CeA. (A) Schematic illustration and the experimental timeline of bilateral CGRP_8–37_ or saline injection in the CeA. (B) Representative images of the microsyringe injection sites, confirming the correct injection of CGRP_8–37_ or saline at bilateral CeA. a: needle tract. Scale bar: 200 μm. (C) Acute CGRP_8–37_ injection transiently alleviated NTG induced mechanical hyperalgesia (*n* = 4 per group; *p* = 0.029). (D) Representative images of rostral-caudal distribution of colocalized pERK- and PKC-δ- positive neurons in the CeA. Scale bar: 100 μm. (E) Schematic illustration of the rostro-caudal anatomical location of CeA relative to the position of bregma. (F) After CGRP_8–37_ injection, the numbers of pERK positive neurons in the CeA were significantly lower than those in the saline group (*n* = 4 per group; *p* < 0.0001). (G) Rostro-caudal distribution of the percentage of pERK positive neurons colocalized with PKC-δ positive neurons in the CeA (left, *n* = 4 per group; − 1.58 mm; *p* = 0.0012; − 1.82 mm; *p* = 0.014); the percentage of pERK/PKC-δ positive neurons of the entire CeA was significantly lower in the CGRP_8–37_ treatment group than that in the saline group (right, *n* = 4 per group; *p* = 0.0001). (H) Rostro-caudal distribution of the percentage of PKC-δ positive neurons colocalized with pERK positive neurons in the CeA (left, *n* = 4 per group; − 1.58 mm; *p* = 0.009); the percentage of PKC-δ/pERK positive neurons of the entire CeA was significantly lower in the CGRP_8–37_ treatment group than that in the saline group (right, *n* = 4 per group; *p* = 0.0026). All data shown are mean ± SEM and analyzed by Mann-Whitney-U test. Significance levels set at *p* < 0.05 *, *p* < 0.01 **, and *p* < 0.001 ***.

**Additional file 7: Supplementary Fig. 7.** The imbalance hypothesis of the PKC-δ activity in the CeA. (A) In this study, we proposed the imbalanced CGRP expression level will sensitize the CGRPR-containing CeA PKC-δ positive neurons along the chronification of migraine. Furthermore, the hyper-activated CGRPR-containing CeA PKC-δ positive neurons plays a critical role in migraine pathogenesis.

**Additional file 8: Supplementary Fig. 8.** Chemogenetic inhibition of the PKC-δ positive neurons in the TG. (A) Schematic illustration of experimental timeline. The TG were dissected on Day 9 and Day 10 as the comparison with or without the CNO application. (B) Representative images of pERK- co-labeled PKC-δ- positive neurons in the TG with or without CNO application (left). The numbers of PKC-δ- positive neurons in the TG were significantly decreased after the CNO application (right, *n* = 4 per group; *p* = 0.002). Scale bar: 100 μm. (C) The percentage of PKC-δ/pERK positive neurons in the TG were significantly decreased after the CNO application (*n* = 4 per group; *p* = 0.022). All data shown are mean ± SEM and analyzed by Mann-Whitney-U test. Significance levels set at *p* < 0.05 *, *p* < 0.01 **, and *p* < 0.001 ***.

**Additional file 9: Supplementary Fig. 9.** The sexual dimorphism after the chronic NTG induction. (A) Schematic illustration of experimental timeline. After 3 weeks of virus expression, PKC-δ-Cre mice were i.p. injected with either vehicle (Ctrl) or NTG (0.1 mg/kg) every other day to day 9 (totally 5 injections) and i.p. injected with CNO (5 mg/kg) on day 10 and 12. The mechanical threshold tested 2 hrs post-treatment after the NTG injection and 1 hr. post-treatment after the CNO injection. (B) Sustained mechanical hyperalgesia alleviated 1 hr. after the CNO application on day 10 and 12 of the male PKC-δ-Cre mice (*n* = 4 per group; *p* < 0.05 *). (C) Sustained mechanical hyperalgesia alleviated 1 hr. after the CNO application on day 10 and 12 of the female PKC-δ-Cre mice (*n* = 4 per group). (D) Insignificant differences of the marbles buried after the chronic NTG injection of the male PKC-δ-Cre mice (*n* = 4 per group). (E) The female PKC-δ-Cre mice buried more marbles after the NTG injection on Day 9 and sustained for two days on Day 11 (*n* = 4 per group; F(1,30) = 12.1; *p* < 0.05 *). All data shown are mean ± SEM and analyzed by Friedman tests with Dunn’s post hoc test (B-D) or Bonferroni post hoc test (E). Significance levels set at *p* < 0.05 *, *p* < 0.01 **, and *p* < 0.001 ***.

**Additional file 10: Supplementary Fig. 10.** Cell counting for the immunofluorescence images. (A) Representative low (left) and high magnification (right) images of rostral-caudal distribution of colocalized pERK/DAPI- and CGRP/DAPI- positive neurons in the TG. Scale bar: 200 μm. (B) Representative low (left) and high magnification (right) images of rostral-caudal distribution of colocalized pERK/DAPI- and PKC-δ/DAPI- positive neurons in the CeA. (C) Representative low (left) and high magnification (right) images of colocalized pERK/DAPI- and SOM/DAPI- positive neurons in the CeA. Scale bar: 100 μm.

**Additional file 11: Supplementary Fig. 11.** Rostral-caudal distribution of colocalized pERK/PKC-δ- and pERK/SOM- positive neurons in the CeA. (A) Representative low (left) and high magnification (right) images of rostral-caudal distribution of colocalized pERK- and PKC-δ- positive neurons in the CeA. (B) Representative low (left) and high magnification (right) images of colocalized pERK- and SOM- positive neurons in the CeA. Scale bar: 100 μm.

## Data Availability

The authors confirm that the data supporting the findings of this study are available within the article and its Supplementary material. Inquiries for additional data are available from the corresponding authors, upon reasonable request.

## Abbreviations

CeA: Central amygdala
CGRP: Calcitonin gene-related peptide
CNO: Clozapine N-oxide
NTG: Nitroglycerin
PBN: Parabrachial nucleus
PKC-δ: Protein kinase c-delta
SOM: Somatostatin
TG: Trigeminal ganglion
